# A Method for the Evaluation of Site-Specific Nephrotoxic Injury in the Intact Rat Kidney

**DOI:** 10.3390/toxics8010004

**Published:** 2020-01-20

**Authors:** Joshua Edwards, Michael Kowal, Aaron VanDreel, Peter Lamar, Walter Prozialeck

**Affiliations:** 1 Department of Pharmacology, College of Graduate Studies, Midwestern University, Downers Grove, IL 60515, USA; plamar@midwestern.edu (P.L.); wprozi@midwestern.edu (W.P.); 2 Department of Emergency Medicine, Captain James A. Lovell Federal Health Care Center, Department of Veteran Affairs, Chicago, IL 60064, USA; Michael.Kowal1@va.gov; 3 Radiology Department, Marshfield Medical Center, Marshfield, WI 54449, USA; vandreel.aaron@marshfieldclinic.org

**Keywords:** drug toxicity, nephrotoxicity, cell viability, amphotericin, gentamicin, indomethacin

## Abstract

In a previously published report we detailed an in situ method to quantify cell death in the renal cortex by perfusing the cell membrane impermeable fluorochrome, ethidium homodimer in situ. The objective of the present study was to use this in situ viability assay to examine cell death following the administration of nephrotoxic drugs known to produce cell death and/or injury in specific segments of the nephron. Male Sprague/Dawley rats were treated with the following nephrotoxicants: Gentamicin, amphotericin-B, and indomethacin. Results of the in situ viability assay indicated that gentamicin and amphotericin-B treatment caused cell death localized in the kidney cortex and medulla, respectively. The urinary biomarker kidney injury molecule—1 (Kim-1) showed significant increases in both gentamicin (20 fold increase) and amphotericin-B-treated (9.2 fold increase) animals. Urinary alpha glutathione-S-transferase (GST) showed significant increases for gentamicin (6.2 fold increase) only and mu GST for amphotericin-B-treated (19.1 fold increase) animals only. These results show that this in situ viability assay provides a sensitive method to identify cell death in different regions of the kidney. Furthermore, urinary alpha GST and mu GST are specific for proximal and distal tubule injury, respectively; urinary Kim-1 demonstrated greater sensitivity to both proximal and distal tubule injury.

## 1. Introduction

Drug-induced nephrotoxicity is common and occurs in 19% to 26% of hospitalized patients [1,2]. Early detection of drug-induced nephrotoxicity is critical as the effect may be reversed if the administration of the offending agent is stopped quickly enough. For example, gentamicin is an often used aminoglycoside antibiotic with well-known clinical nephrotoxic effects, for review see [3].

The use of urinary biomarkers to detect early-stage nephrotoxicity is an important aspect of general practice medicine, preclinical drug safety testing and workplace monitoring programs [4]. While the presence of some of these biomarkers in urine is not necessarily due to renal damage, there are urinary biomarkers that purportedly indicate specific sites of nephrotoxic injury. Biomarkers such as kidney injury molecule-one (Kim-1) [5] and alpha glutathione S-transferase (GST) are elevated in urine when proximal tubules become damaged or injured. Renal papillary antigen 1 (RPA-1) and mu GST are biomarkers for renal papillae and distal tubules, respectively. In contrast to the use of non-invasive serum or urinary biomarkers to determine renal injury, more traditional methods involve the histological examination of renal tissue to detect cell death or injury.

Previous reports have described the use of the cell-membrane impermeable DNA-binding fluorochrome, ethidium homodimer, to detect changes in cell viability in a variety of tissues such as kidney [6] and lung [7]. As part of our ongoing research to identify the mechanisms by which the environmental toxicant, cadmium, produces its nephrotoxic effects we have developed a method to assess changes in cell viability in the renal cortex. Briefly, the intact kidney of an anesthetized rat is perfused with ethidium homodimer following acute mercuric mercury or gentamicin treatment [6]. This method of detecting renal cell death in the cortex has the potential to also determine drug-induced cell death in other parts of the kidney including the medulla and papilla.

The objective of the present study was to compare the ability of site-specific urinary biomarkers of renal injury to a viability assay utilizing an in situ perfusion to detect changes in renal cell viability. Well-established site-specific nephrotoxic drugs were administered to rats at doses and treatment regimens reported in the literature to cause renal dysfunction and toxicity at various sites within the kidney specifically; gentamicin (cortex), indomethacin (papilla), and amphotericin (medulla). Tissue sections from the three regions of the kidney were examined in parallel with changes in cell viability assessed by an in situ assay involving ethidium homodimer labeling in the cortex, medulla, and papilla of the kidney. In addition, urine samples were collected to examine changes in renal function as well as levels of urinary site-specific biomarkers.

## 2. Materials and Methods

### 2.1. Treatment of Animals

Unless otherwise noted, all reagents were purchased from Sigma (St Louis, MO, USA). Adult male Sprague-Dawley rats (Envigo, Indianapolis, IN, USA) were treated with one of three commonly used drugs using dosing regimens reported in the literature to cause nephrotoxicity. Gentamicin-treated animals received once daily injection for 8 consecutive days at a daily dose of 100 mg/kg, i.p [8]. Indomethacin was given as a single dose (20 mg/kg) by oral gavage [9]. Lastly, amphotericin B (with the solubilizing agent deoxycholate) was given by once daily injections for 5 consecutive days at a dose of 15 mg/kg, i.p. amphotericin B [10]. All drugs were dissolved in isotonic saline (vehicle) and then sterile filtered with 0.22 µm filters. Control animals were dosed in the same manner for each respective drug dosing regimen with saline vehicle alone. Control-treated animals in the amphotericin B experiments were injected with saline and corresponding amounts of deoxycholate. *n* = 8 for each drug treatment and the respective control group. Following the last dose, animals were placed in individual metabolic cages for 24 h for urine sample collection. The urine volume was measured and samples aliquoted and stored at −80 °C for later analysis. All animal experiments and procedures were reviewed and approved by the Midwestern University Institutional Animal Care and Use Committee and performed in an animal facility accredited by the Association for Assessment and Accreditation of Laboratory Animal Care International (protocol # 1500; approved from June 2008 through April 2017).

### 2.2. Quantification of Urinary Biomarkers of Renal Injury

Aliquots from 24 h urine samples were analyzed for protein using the Bradford Coomassie blue dye assay (Thermo Fisher cat. # 23236) and creatinine according to the methods of [11]. Urine samples were also analyzed for the following biomarkers of renal injury: Kim-1 (R&D Systems, Minneapolis, MN, USA; cat # DY3689), alpha-GST (Biotrin International Ltd., Dublin, Ireland; cat # BIO64RT), mu-GST (Biotrin International Ltd., Dublin, Ireland; cat # BIO76YB1) and RPA-1 (Argutus Medical Ltd., Dublin, Ireland; cat # BIO89RPA1).

### 2.3. Renal Perfusion

At the end of the various drug treatment protocols, rats were injected intraperitoneally with ketamine/xylazine (67/7 mg/kg). After the opening of the abdominal cavity, the left kidney was exposed by isolating it from the surrounding connective tissue. The abdominal cavity was opened and the left kidney was isolated from surrounding connective tissue. The aorta was freed from the surrounding connective tissue and from the adjacent vena cava. A 4.0 silk ligature (Roboz Surgical, Gaithersburg, MD, USA) was positioned around the aorta as far caudally as possible (approximately 2 cm below left renal artery) and tied immediately. A second ligature was positioned around the aorta just above the left renal artery and tied just prior to the insertion of the catheter. In order to allow for an isolated perfusion of the left kidney, a third ligature was tied around the right renal artery and vein to prevent perfusion of the right kidney, just prior to insertion of the perfusion catheter. The perfusion catheter was fashioned from a 23 gauge stainless steel needle and connected to polyethylene (PE 50) tubing filled with a solution of 5 μM ethidium homodimer in physiological saline solution (PSS) which contained (mM): 115.0 NaCl, 5.5 glucose, 16.0 NaHCO_3_, 1.0 MgCl_2_, 0.2 Na_2_HPO_4_, 0.8 NaH_2_PO_4_, 1.0 CaCl_2_, and 5.0 KCl. An incision was made approximately half way through the aorta just above the lower ligature and the catheter was inserted into the aorta and advanced to a position just below the left renal artery. The catheter was tied and secured in place with a new ligature. Once the catheter was secured, ethidium homodimer was perfused with a peristaltic pump at a flow rate of 1 mL/min for 5 min then increased to 3 mL/min for 5 min. The pressure of the perfusate was 50 mmHg at a flow rate of 1 mL/min and 100 mmHg at 3 mL/min, as measured in preliminary experiments with a low pressure transducer (Grass P10EZ) attached to a Model 7 Grass polygraph (Grass Technologies/Astro-Med, Inc., West Warwick, R.I.). The left kidney was then perfused with PSS at 3 mL/min for 10 min to wash out any residual or unbound ethidium homodimer. The perfused left kidney was removed, decapsulized, cut through the transverse plane into three sections and immediately frozen separately at −80 °C for later cryosectioning. Animals were then euthanized by exsanguination and pneumothorax while under anesthesia [6].

### 2.4. Visualization of Necrotic Cells

The frozen kidney samples were cryosectioned and processed according to the methods of [6]. Briefly, kidneys were cryosectioned at five microns and mounted on glass slides. After fixation/permeabilization in −20 degree Celsius methanol, 0.3 µM 4’6-diamidino-2-phenylindole (DAPI) was applied to the sections in order to visualize all nuclei. An aqueous mounting medium-AquaPolymount (Warrington, PA, USA)-was then used to mount the sections under glass coverslips. Within 24 h, labeled sections were viewed using a 40× objective under phase contrast and fluorescent illumination on a Nikon Eclipse 400 fluorescent microscope. Total nuclei and ethidium-labeled nuclei were quantified within randomly-selected fields. There were approximately 200−300 cells in an area of 9.1 × 10^4^ µm^2^ in each field. Viability analysis was performed on digital images from three fields per slide, four slides per animal. Digital images were captured with a 5.0 megapixel Evolution^TM^ MP digital camera by Media Cybernetics. Previously, we noticed that the staining intensity of ethidium homodimer and especially DAPI faded over time [6]. Therefore all the images of renal sections were captured within 48 h following DAPI staining. Digital images were processed using Image Pro Plus Software (v. 7.0) (Media Cybernetics, Silver Spring, MD, USA, 2009).

### 2.5. Kidney Histopathology Scoring

Blinded studies examining histopathologic changes in the renal cortex of gentamicin-treated and control rats were performed to verify the in situ viability assay, urinalysis and biomarker findings. Samples only from gentamicin-treated animals were used because that treatment resulted in the largest change in cell viability and renal function. Following the methods of Rouse et al. [12]; H&E stained sections were scored based on a composite of degeneration, necrosis, tubular dilation, protein casts, apoptosis and regeneration: 0 = no damage; 1 = degeneration only; 2 ≤ 25%; 3 = 25%−50%; 4 51%−75%, and 5 > 75% of tubular cells affected. A trained technician blinded to treatment group scored H&E sections as described above; tissue sections were scored from 3 different animals from either treatment group.

### 2.6. Statistical Analysis

Data were analyzed using the Graph Pad Prism^®^ statistical program (v. 8.0) (La Jolla, CA, USA, 2018). One-way or two-way analysis of variance (ANOVA) was utilized where appropriate to determine statistical differences. A post-hoc Sidak’s test was performed to determine which mean values were significantly different from control values if significant differences between sample means were found (*p* < 0.05).

## 3. Results

No animals died during this study following treatment with of the site-specific drug dosing regimens. Upon dissecting the non-perfused kidney, pale and or discolored areas were noted in gentamicin and amphotericin-B-treated animals.

### 3.1. Dosing of Site-Specific Nephrotoxicants Caused Histological Changes in Kidney Tissues

**Figure 1:**
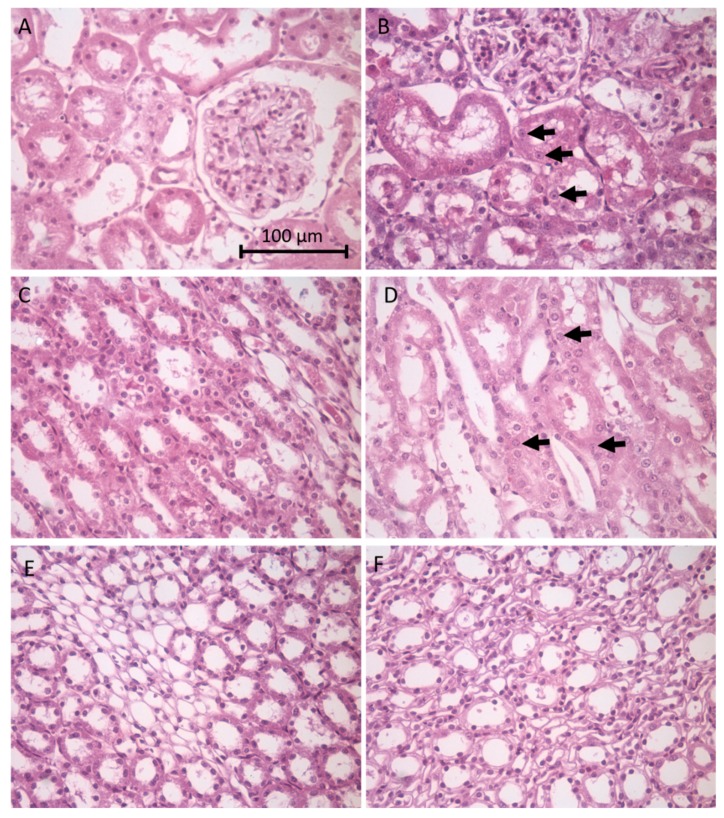
Histological changes in the different regions of the kidney following treatment of site-specific nephrotoxicants. Following treatment, kidney tissue was collected and later sectioned to 5 µm thick sections, then stained with hematoxylin and eosin. Images are representative images from male Sprague/Dawley rats treated with the proximal tubule/cortex nephrotoxicant gentamicin (100 mg/Kg/day i.p. for 8 days). H&E histological sections of renal cortex are shown in panels (**A**) (control) and (**B**) (treated). The distal tubule/medulla toxicant amphotericin B (15 mg/Kg/day i.p. for 5 days), renal medula is shown in panels (**C**) (control) and (**D**) (treated). Lastly, collecting duct/papilla nephrotoxicant indomethacin (single dose 20 mg/Kg p.o.), renal papillae is shown in panels (**E**) (control) and (**F**) (treated). Arrows indicate nuclear condensation, indicative of apoptosis. Scale bar in panel A is 100 microns in length; all images are of the same magnification.

Histological changes were examined in the kidney from nephrotoxicant- and control-treated animals. The cortex, medulla and papilla were examined for evidence of apoptotic or necrotic cell death and for morphological changes in the kidney tubules. As shown in Figure 1B tubule epithelial cells in the renal cortex show evidence of apoptosis with nuclear condensation (arrows) in gentamicin-treated (100 mg/kg/day, i.p. for 8 days) animals. Similar nuclear condensation is shown (arrows) in renal epithelial cells in the medulla following amphotericin B-treatment (15 mg/kg/day, i.p. for 5 days) as shown in Figure 1D. Figure 1F shows less overt tubular damage in the papilla in indomethacin-treated animals (20 mg/kg single dose p.o.). Blinded histopathology scores of the renal cortex from control and gentamicin-treated rats showed significant damage in terms of general tubular degeneration, tubular dilation, necrosis, apoptosis with an average score of 2.6 ± 0.4 for gentamicin treatment and an average of 0 for control animals; scoring range was 0–5.

### 3.2. The In Situ Viability Assay Showed Significant Changes in Cell Death

**Figure 2:**
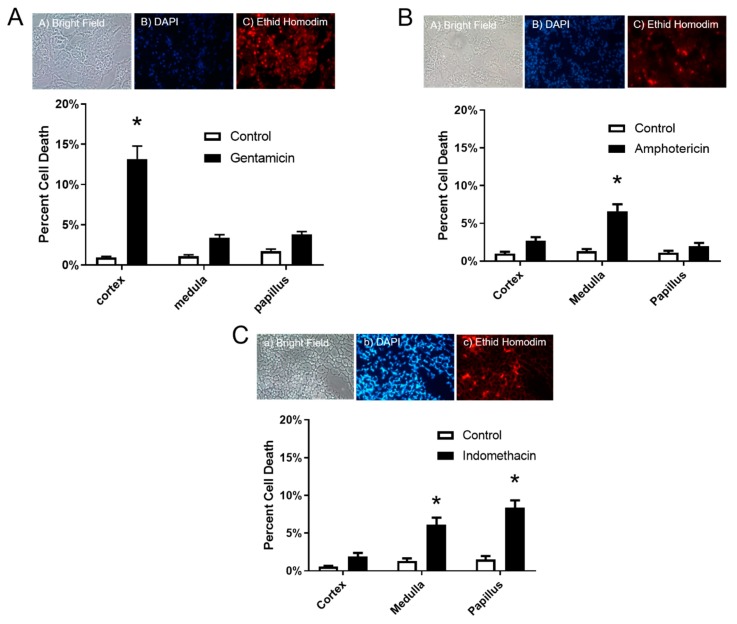
Effects of various site specific nephrotoxicants on cell membrane permeability in the renal cortex, medulla and papilla. Male Sprague/Dawley rats were treated with a proximal tubule/cortex nephrotoxicant (gentamicin 100 mg/Kg/day i.p. for 8 days) a distal tubule/medulla toxicant (amphotericin B 15 mg/Kg/day i.p. for 5 days) and collecting duct/papilla nephrotoxicant (indomethacin single dose 20 mg/Kg p.o.). Control animals were treated similarly with vehicle alone (saline). Data show percent ethidium homodimer labeled (dead) cells in the cortex, medulla and papilla for (**A**) gentamicin, (**B**) amphotericin B, and (**C**) indomethacin. Figure insert shows representative images of the same field of view showing a) bright field, b) DAPI staining labelling all nuclei, and c) ethidium homodimer labeling in the cortex of gentamicin-treated animals, medulla of amphotericin B treated and papillae of indomethacin treated animals. An asterisk (*) indicates statistically significant differences from vehicle alone control (two-way ANOVA followed by Sidak’s multiple comparison test; *p* ≤ 0.05, *n* = 6 for all groups).

Results of the in situ cell viability assay showed a significant increase of 13.1% in cell death in the cortex only after gentamicin dosing (Figure 2A), and 6.6% increase in the medulla after amphotericin B dosing (Figure 2B). Lastly, Indomethacin dosing resulted in an increase in cell death in both the medulla and papilla of 6.1% and 8.4%, respectively (Figure 2C).

### 3.3. Site-Specific Nephrotoxicants Caused Changes in Urine Volume, Protein, and Creatinine

Treatment with gentamicin resulted in changes in urinary parameters characteristic of renal injury including polyuria and proteinuria, see Table 1. Amphotericin B dosing caused significant decreases in urinary creatinine; while, indomethacin treatment did not result in any statistically significant change, see Table 1.

Most experimental animal studies or clinical trials report urinary biomarkers on a per mg urine creatinine basis. As shown in Table 1, amphotericin dosing resulted in a significant decrease in urinary creatinine level. As such, urinary biomarker data expressed on a per mg creatinine basis would artificially inflate biomarker values for amphotericin-treated animals. To avoid this error, all biomarker data are expressed on a per Kg bodyweight per urine volume collected over 24 h.

### 3.4. Site-Specific Nephrotoxicants had Differential Effects on Levels of Urninary Biomarkers of Renal Injury

**Figure 3:**
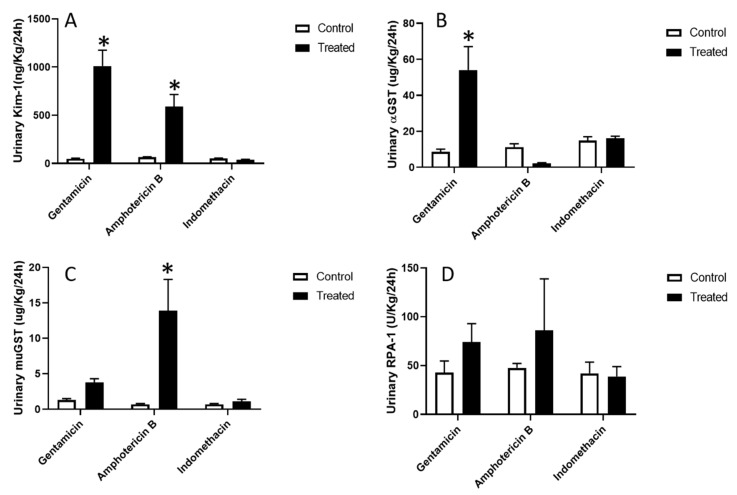
Changes in urinary biomarkers following exposures to site specific nephrotoxicants. Male Sprague/Dawley rats were treated with a proximal tubule nephrotoxicant (gentamicin 100 mg/Kg/day i.p. for 8 days) a distal tubule toxicant (amphotericin B 15 mg/Kg/day i.p. for 5 days) and collecting duct nephrotoxicant (indomethacin single dose 20 mg/Kg p.o.). Control animals were treated similarly with vehicle alone (saline). Animals were placed in metabolic cages for 24 h following treatment and urine collected. Data are changes in urinary levels of (**A**) Kim-1, (**B**) αGST, (**C**) mu GST, and (**D**) renal papillary antigen 1 (RPA-1) normalized to Kg body weight and ml urine volume collected for 24 h. An asterisk (*) indicates statistically significant differences from vehicle alone control (two-way ANOVA followed by Sidak’s multiple comparison test; *p* ≤ 0.001, *n* = 6 for all groups).

Urinary levels of Kim-1 were significantly elevated by a 20 and 9 fold increase following gentamicin and amphotericin B dosing, respectively (Figure 3A). Alpha-GST is a biomarker specific to proximal tubule epithelial cells with a 6 fold increase in the urine of gentamicin-treated animals (Figure 3B). The distal tubule biomarker, mu-GST was significantly elevated with a 19 fold increase in the urine of amphotericin B-treated animals (Figure 3C). However, the biomarker for collecting ducts, RPA-1, did not show any significant changes in urinary levels in any of the treatment groups (Figure 3D).

## 4. Discussion

In this study, we found histological changes of tubule epithelial cells indicating epithelial cell death in the renal cortex of gentamicin-treated (100 mg/kg/day, i.p for 8 days) animals and in the medulla of amphotericin B-treated (15 mg/kg/day, i.p. for 5 days) animals. In agreement with these findings, the in situ viability assay using ethidium homodimer showed that gentamicin caused a significant increase in labelled dead cells in the renal cortex and a significant increase in cell labelling in the medulla of amphotericin B-treated animals. However, the in situ viability assay indicated increased cell death in indomethacin-treated animals in the medulla and papillae while these animals showed no overt histopathological changes. Furthermore, significant changes in indicators of renal function (i.e., polyuria and proteinuria) were found in gentamicin-treated animals and changes in urinary creatinine were found in amphotericin B-treated animals. We also report significant increases in urinary Kim-1 levels following treatment with either gentamicin or amphotericin B. While urinary alpha GST was only increased following gentamicin treatment and only mu GST was increased after amphotericin-B treatment. Overall, the results shown here indicate that the in situ viability assay is a more sensitive, but also more invasive and labor intensive, means to determine renal injury following site-specific drug-induced nephrotoxicity.

A drawback of the in situ viability assay used here is that regions of the kidney were examined for cell death instead of actual segments of the nephron (i.e., proximal, distal tubules and collecting ducts). For example, within the cortex of the kidney, there will be a combination glomerular cells with epithelial cells from both proximal and distal tubules. However, the majority of epithelial tubule cells will be from proximal tubules. Therefore ethidium homodimer labelled cells in the cortex are most likely that of proximal tubule epithelial cells. By extension, it would be difficult to distinguish cell death between the three segments (S1, S2, and S3) of the convoluted proximal tubule. It is feasible that cell death could be quantified in each specific segment of the nephron if ethidium homodimer labelled tissue sections are co-labelled for specific markers of proximal tubules (e.g., aquaporin 1) and collecting ducts (e.g., aquaporin 2) [13].

One of the most significant findings from the present study was that the results of the site-specific biomarkers experiments were consistent with the results of the histopathological and ethidium labelling studies. For example, alpha GST and Kim-1, which are primarily markers of proximal tubule injury were most elevated in urine from animals that were treated with proximal tubule nephrotoxicant gentamicin. Kim-1 was also elevated in the urine from amphotericin B-treated animals but was not altered with indomethacin treatment. Together, these results provide further evidence that Kim-1 and alpha GST are highly sensitive makers of proximal tubule injury.

One confounding factor in extrapolating the experimental findings presented here to clinical practice is that chronic or acute disease conditions not necessarily related to nephrotoxic drug dosing may alter biomarkers, for review see [14]. For example, sepsis alone may cause renal injury with subsequent antibiotic dosing exacerbating the kidney injury [15]. Another example is diabetic kidney disease, the prevalence of which is increasing in proportion to persons with diabetes despite increased use of blood glucose-lowering medications [16].

In conclusion, the in situ renal cell viability assay used here was shown to be sensitive and robust enough to detect cell death in various gross regions of the kidney. This assay may prove useful in a wide variety of toxicological studies involving toxicants that cause cell death in different segments of the nephron. Further effort is needed to identify and develop more sensitive site-specific biomarkers to detect renal injury or cell death in specific segments of the nephron. MicroRNAs found in urine have the potential to fill this important gap in the development of more sensitive biomarkers of site-specific renal injury [17].

## Figures and Tables

**Table 1:** Changes in urinary parameters after acute nephrotoxic doses of gentamicin, amphotericin and indomethacin.

Treatment	Urine Volume (mL)	Urinary Protein (mg/kg/24 h)	Urinary Creatinine (mg/kg/24 h)
Gent.–Control	15.8 ± 0.9	39.5 ± 2.9	40.1 ± 2.6
Gent.–Treated	* 34.2 ± 4.5	* 126.8 ± 16.4	48.6 ± 6.1
Amph.–Control	18.3 ± 1.7	49.8 ± 7.5	36.8 ± 2.0
Amph.–Treated	23.2 ± 3.4	63.1 ± 14.4	* 23.1 ± 2.5
Indo.–Control	16.0 ± 1.6	36.2 ± 5.2	42.5 ± 4.7
Indo.–Treated	13.6 ± 1.8	45.7 ± 3.9	35.7 ± 2.4

Male Sprague Dawley rats were given nephrotoxic dosing regimens of gentamicin, amphotericin B or indomethacin then placed in metabolic cages for 24 h for urine collection. Control animals were treated similarly with vehicle only (saline). Asterisk (*) indicates statistically significant differences between saline-treated control animals (two-way ANOVA followed by post-hoc Sidak’s multiple comparison test, *p* ≤ 0.05, *n* = 6 for all treatment groups).
